# Design, processing, and modeling for longitudinal multiomics microbiome data

**DOI:** 10.3389/fcimb.2026.1837109

**Published:** 2026-08-20

**Authors:** Kaiyan Ma, Margaret Thairu, Kris Sankaran

**Affiliations:** 1 Changping Laboratory, Beijing, China; 2 Wisconsin Institute for Discovery, University of Wisconsin - Madison, Madison, WI, United States; 3 Department of Statistics, University of Wisconsin - Madison, Madison, WI, United States

**Keywords:** data integration, interaction modeling, longitudinal data, microbiome, multiomics, networks

## Abstract

Longitudinal multiomics studies can reveal mechanisms underlying microbiome dynamics. Though gathering such data has become increasingly accessible, challenges remain in experimental design, data processing, and interaction modeling. This mini-review surveys practical approaches for analyzing longitudinal multiomics microbiome data. We provide an overview of fundamental questions these experimental designs can address, discuss concepts for reducing confounding, review tools for data management, and describe statistical and machine learning methods for identifying interactions across time and biological layers. We conclude with emerging trends and open problems.

## 1 Introduction

Microbes exist within communities of rich taxonomic and metabolic diversity, interacting through metabolic exchange, competition, and cooperation. Through these interactions, microbial communities mediate fundamental ecological processes, from nutrient cycling to transformation of environmental conditions. Within a host, these interactions grow in complexity, with the microbiome and its host shaping one another’s physiology, function, and health. Understanding both scales of interaction – within the microbial community and between microbes and their hosts – is a central goal of microbiome research.

Advances in high-throughput molecular approaches have enabled increasingly detailed characterization of microbial ecosystems. Techniques such as 16S rRNA gene sequencing and metagenomics are widely used to characterize microbial community composition, while functional “-omics” approaches – including metabolomics, proteomics, lipidomics, transcriptomics, and epigenomics – provide insight into the molecular activity of both microbial communities and their hosts. When multiple such layers are gathered jointly, the resulting data are multiomic, and multiomic studies aim to understand interactions across layers and infer the mechanisms driving biological phenotypes. These goals depend on integration, which we define as merging multiomic data into a single framework where omic layers can be compared and their interactions uncovered.

Multiomic data are increasingly common, yet the development and application of robust statistical methods for their integration has lagged behind. Traditional statistical approaches fail to account for key properties of microbiome datasets, like sparsity and high dimensionality (Deek et al., 2024). These challenges are magnified in longitudinal studies, where samples exhibit strong temporal dependence and effects may cascade. Despite these challenges, longitudinal data can offer rich insight into the mechanisms driving phenotypes of interest [e.g (Zhou et al., 2019b; Lloyd-Price et al., 2019; Bai et al., 2024; Gong et al., 2025; Creus-Martí et al., 2025)].

**Figure 1:**
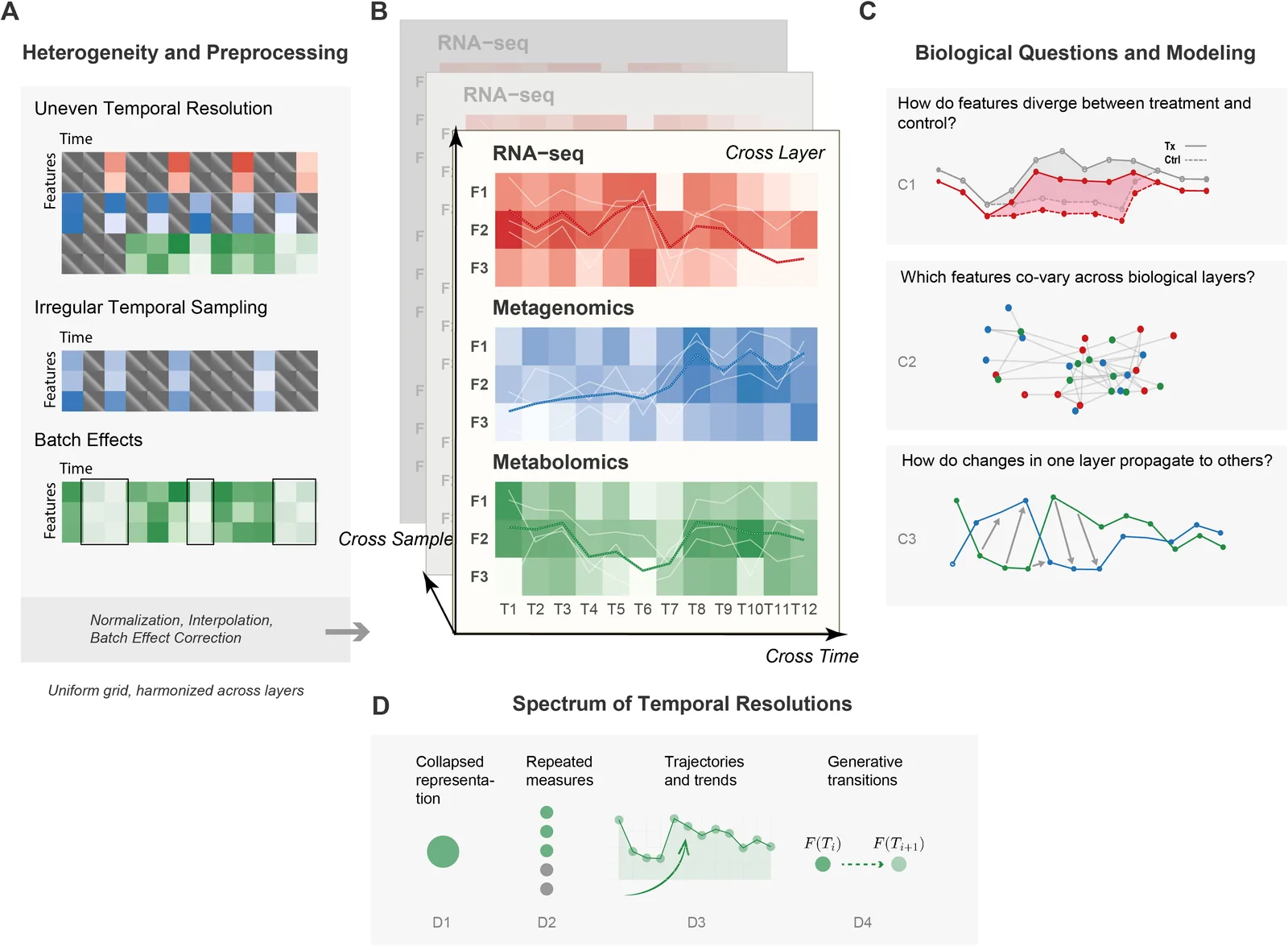
A summary of methods used for processing and modeling longitudinal multiomics data in microbiome studies. **(A)** Preprocessing: Challenges include irregular temporal sampling designs, alignment across multiomics layers, batch effects, sparsity, and skewness issues. **(B)** Data Integration: Processed datasets are integrated across three axes: cross-layer (*inter*-omics/features), cross-sample (*inter*individual), and cross-time (longitudinal). **(C)** Modeling Objectives: Methods target specific biological goals, ranging from identifying temporal shifts in feature distributions to inferring cross-layer associations and causal mechanisms. **(D)** Temporal Resolutions: Modeling approaches rely on diverse abstractions and assumptions for longitudinal data: (1) Collapsed representations, (2) Repeated measurements, (3) Trajectories, and (4) Generative dynamics.

Recent reviews cover longitudinal microbiome analysis (Silverman et al., 2018; Kodikara et al., 2022; Lyu et al., 2023) and multiomics integration (Chetty and Blekhman, 2024; Baião et al., 2025; Muller et al., 2026). Martínez Arbas et al. (2021) additionally survey measurement strategies for longitudinal microbiome studies and argue for their wider adoption. Recently, Sherwani et al. (2025) systematically review longitudinal integration methods for microbiome data and quantify trends in their development and use. Neither treats the statistical and machine learning issues that arise when longitudinal and multiomics aspects are combined, from experimental design and processing to modeling and interpretation (**Figure 1**). This review fills that gap, treating methods in depth and assuming no prior background. Section 2 discusses statistical characteristics of multiomics data. Section 3 defines key terms and offers case studies illustrating the scientific questions that can be answered in this setting. Sections 4–5 trace the data analysis workflow for rigorously answering them. Section 6 considers emerging trends and open problems.

## 2 Statistical characteristics of microbial multiomics data

Multiomics integration is challenging because each layer possesses unique statistical properties that require specialized handling. Compositionality, zero-inflation, high-dimensionality, and distributional assumptions vary across layers (**Table 1**). Ignoring these differences results in errors that propagate through downstream integration and yield biologically inaccurate conclusions (Baião et al., 2025).

**Table 1:** Summary of statistical characteristics across multiomic layers.

Meta-omic layer	Data characteristics	Missing data/zeros	Hierarchical structure	Key unique challenges
16S rRNA Amplicon				
	Compositional and sparse: discrete counts relative to library size.	Structural zeros (biological absences) and sampling zeros (depth-driven).	Non-independent across taxonomic ranks (Phylum → Species) and phylogeny.	PCR bias & limited resolution: primer gaps distort abundance; short reads limit species-level ID (Rathod and Silverman, 2026).
Metagenomics				
	Compositional and overdispersed: higher complexity than 16S; extreme sparsity in rare taxa.	Structural zeros (biological absences) and sampling zeros (depth-driven).	Genes map to both specific pathways and taxonomic bins.	Reference gaps: misassignment due to incomplete reference genomes/MAG databases (Chen et al., 2020).
Metatranscriptomics				
	Compositional and sparse: counts influenced by both species abundance and gene regulation; can be more sparse than metagenomic data.	Structural zeros (biological absences and genes in “off” state), sampling zeros (depth-driven), and technical dropouts.	Nested hierarchy: Gene → Pathway → Taxon.	Normalization dependency: cannot interpret expression without metagenomic-based community structure correction (Zhang et al., 2021; Cho et al., 2023).
Metaproteomics				
	Log-normal (post-translational): high sparsity due to MS acquisition limits.	High rate of technical zeros for low-abundance signals below detection limits.	Peptide → Protein → Taxon (proteogenic redundancy).	Database complexity: requires metagenomic-derived databases for accurate peptide identification (Nebauer et al., 2024; Arıkan and Atabay, 2024).
Metabolomics				
	Continuous and right-skewed.	Technical zeros (signal below limit of detection) and biological absence.	Chemical classes and metabolic reaction networks.	Annotation & attribution: large “dark matter” gap in references; difficult to assign to host vs. microbe (da Silva et al., 2015; Peisl et al., 2018).

Common challenges across all multiomics layers, including high dimensionality, batch effects, multiple testing burden, hierarchical community structure, and normalization. These issues impact statistical approaches and cross-layer integration.

Compositionality: A key characteristic of sequencing-based -omics is compositionality - the fact that the total number of reads is limited not by the abundance of microbes in the sample, but by the sequencing depth. Data collected by these methods do not represent the absolute counts of microbes, but rather their relative proportions. This leads to spurious correlations where the relative abundances of all microbes appear linked, even if the absolute abundances of individual microbes change (Gloor et al., 2017). In 16S amplicon and metagenomic data, compositionality influences the results at a single level: the taxonomic level. Metatranscriptomic data however, are constrained by compositionality at two distinct levels: the shifting proportions of the microbial community members and the differences in each species’ transcriptional regulation (Cho et al., 2023). Metaproteomic data are similarly constrained at two levels, with the instrument detection limit acting as a second compositional filter in which high-abundance proteins mask lower-abundance ones (Van Den Bossche et al., 2025). Accounting for the ways compositionality can appear across microbiome multiomics layers has been an area of active interest and will continue to require careful study as new omics modalities emerge, as discussed in Section 6. **Supplementary Table 2** summarizes how methods account for read depth and compositionality.

Zero-Inflation and Missing Data: Zero-inflation is a defining property of microbial multiomics data. Identifying the source of these zeros informs the assumptions for later modeling or imputation. There are three primary types:

1. *Structural zeros* arise when a signal is truly absent from the system – a taxon not present in the environment, a gene or protein not expressed under the given conditions.
2. *Sampling zeros* occur when a feature is present in the sample but undetected because of sequencing depth or library size (e.g., a low-abundance microbe falling below the detection threshold). These zeros are stochastic and due to undersampling.
3. *Technical zeros* result from failures in the experimental pipeline, such as RNA degradation, poor protein extraction, or masking effects in mass spectrometry (MS).

All multiomics layers contain a mixture of these types of zeros, though the relative contributions differ (**Table 1**). In sequencing-based assays (16S, metagenomics, and metatranscriptomics), zeros are driven primarily by variation in sequencing depth, though metatranscriptomics additionally suffers from technical dropouts from RNA degradation. In MS-based approaches (metaproteomics and metabolomics), technical zeros dominate, arising from instrument detection limits rather than biological absence. When considering how to handle these zeros, sampling zeros are candidates for imputation, structural zeros should not be imputed, and technical zeros require separate quality control steps before modeling.

High Dimensionality and Distributional Assumptions: All multiomics layers share the challenge of high dimensionality, where the number of features *p* greatly exceeds the number of samples *n*. Dimensions vary widely across layers, from hundreds of annotated metabolites to millions of genomic variants. Features often have hierarchical structures (e.g., taxonomic or functional trees), and distributional assumptions vary by data type: 16S amplicon, metagenomic, and metatranscriptomic data typically follow overdispersed count distributions, while metabolomics and metaproteomics data are treated as continuous. Many cross-layer approaches use dimensionality reduction and data integration methods to address these issues of feature imbalance and high dimensionality (Baião et al., 2025).

## 3 Integration phases

Many of the challenges in longitudinal multiomic microbiome analysis stem from the difficulty of standardizing data collection. For example, outside highly controlled experiments, exactly uniform temporal spacing is impossible, yet many statistical techniques assume such uniformity. Similarly, cost differences make it unwise to assay all biological layers at each timepoint, so sampling resolution varies across layers, but traditional methods assume complete data across all biological layers for each sample.

In practice, a central choice in multiomics analysis is whether to learn relationships after preliminary modeling steps (late integration) or instead apply a single statistical framework to study multiple layers at once (early integration). Pattaroni et al. (2025) provides an example of late integration. Here the authors study how airway-associated microbial communities and immune responses develop in early childhood. They followed 32 children for one year, characterizing the bacterial, fungal, and viral microbiome profiles from the upper airway along with host transcriptomics from nasal epithelial cells and peripheral blood. They first independently analyzed each omic layer to identify age-associated temporal trends, like shifts in microbial taxa composition and changes in immune gene expression over time. The authors then compared these trajectories across datasets to identify correlations between microbial colonization patterns and host immune responses. This analysis revealed associations between the abundance of specific microbial taxa and the activation of immune transcriptional pathways. Such an approach has the advantage of modularity – layer-specific methods can be easily interchanged. However, since each layer is modeled independently, the assumptions linking them remain implicit, making cross-layer mechanistic claims difficult to establish.

Early integration, on the other hand, considers all data within a single mathematical framework. Zhou et al. (2024) provide an example involving early-integration components. The authors sought to understand the link between the microbiome and glucose regulation. They collected samples from four body sites (oral, nasal, skin, and gut) from 86 adults over a six-year period. The resulting data included proteomics, metabolomics, lipidomics, and clinical and cytokine markers. Rather than analyzing each data type separately, the authors integrated these datasets by estimating a correlation network of subsets of modalities and Bayesian negative-binomial longitudinal mixed-effects models that inferred interactions across molecular layers (see Section 5). This early integration identified site-specific microbiome–cytokine interaction networks that influenced microbial community structure. Notably, the resulting networks suggested that microbial taxa were more strongly associated with specific host molecular signals than with body site location itself. By modeling cross-layer dependencies directly, this approach highlighted potential mechanistic interactions between host immune signaling and microbial community structure that late integration would be unlikely to detect.

Together, these studies illustrate the trade-offs between analytical integration strategies in longitudinal microbiome multiomics. Late integration analyses are often simpler to implement and can reveal meaningful correlations across biological layers, particularly when datasets differ substantially in sampling frequency or completeness. In contrast, early integration approaches can provide improved modeling coherence and insight into cross-layer dependencies, but they require more sophisticated statistical frameworks and careful handling of missing data, high dimensionality, and temporal structure.

Although late and early integration are helpful anchors for practical analysis, they are only two points along a wider integration spectrum. For example, intermediate phase integration learns a separate representation of each layer while sharing information across layers. In this spirit, Lock and Dunson (2013) estimate separate clusters per layer but link them through a joint consensus clustering. Representations can also be directly learned in a shared latent space. The encoding step respects the structure of each omics layer, but the representations themselves are learned jointly. This can be done with matrix factorization, autoencoding architectures, or multimodal embedding methods. A related methodological literature addresses the challenge of incomplete data, where integration must handle partially observed modalities, a task called mosaic integration. These approaches typically adapt factorization or representation learning to the missing-modality setting. For example, Park et al. (2025) define an encoder that projects any subset of layers into a common latent space and applies it to genomics and proteomics data in Alzheimer’s disease. More recently, biological foundation models, trained across heterogeneous experiments, have become another avenue to shared representations for mosaic data.

Integration phase is a key choice in any multiomics analysis. For longitudinal data, a second, largely independent choice is whether a method is time-aware. Does it recognize that samples from the same host are drawn sequentially, and does it support reasoning about the specific dynamics within and across layers? We discuss the forms this can take in Section 5.

## 4 Data collection and management

Care taken in initial data management and processing pays off during modeling and analysis. Effective experimental designs avoid confounding, appropriate data structures support reproducibility, and accounting for technical effects during measurement increases power to detect biological signals. Few data management and processing methods have been proposed specifically for longitudinal multiomics data, but combining methods developed separately for longitudinal and multiomics contexts gives a practical path forward.

### 4.1 Experimental design

Cross-sectional, single-omic microbiome studies already involve important design parameters, like sample size, replication, library size, and positive and negative controls. Longitudinal, multiomics studies add further complexity, like temporal sampling density, which biological layers to assay at each timepoint, and potentially treatment timing. Despite this complexity, a few principles guide many design decisions.

Blocking is the practice of assigning competing treatments to samples with similar baseline variation (Meier, 2022). For example, having the same study participant undergo several treatments (or both treatment and control) is often better than assigning the treatments to separate groups, because it improves sensitivity where treatment effects are consistent but masked by high participant-to-participant heterogeneity (Vandeputte et al., 2021). Blocking is especially powerful in longitudinal microbiome studies, where alternate treatment and control regimes can be assigned sequentially to the same participants (Jeganathan et al., 2018; Zhou et al., 2024). Beyond blocking, sampling times should align closely with the relevant biological state. In studies measuring microbiome change after medical treatment or surgery, samples collected at different timepoints may capture post-treatment recovery dynamics rather than the intervention’s actual effects. These practices help minimize the impact of participant-to-participant differences, but some heterogeneity remains. The remaining heterogeneity can be addressed in part through randomization, which refers to assigning treatment and design parameters with a random number generator (Ding, 2023) to the study design. In practice, though samples are collected longitudinally, they should be extracted and sequenced in random order. Otherwise, true temporal trends become impossible to disentangle from technical changes in the sequencing workflow.

Beyond randomization and blocking, controls and replication enable correction of technical effects introduced during measurement (Glass, 2014). Without these samples, correction methods must rely on strong assumptions (see Section 4.3). In sequencing assays, negative controls are samples that contain no biological material but are processed through the entire DNA extraction, library preparation, and sequencing workflow. These “extraction blank” samples reveal systematic contamination and effects from reagents, operator handling, and lab instruments (Fierer et al., 2025). Negative controls identify technical artifacts but cannot determine whether an experiment was sensitive enough to detect true biological signals. Positive controls address this. Across plates, wells can be reserved for spike-in controls or mock communities (Galla et al., 2023; Colovas et al., 2022). In a longitudinal setting, controls should span the full sampling duration, not just be taken at baseline. A running series of controls allows detection of technical effects that emerge over time. For example, samples gathered early and late in a study may differ simply because they were stored for different lengths of time. In metabolomics specifically, mixture positive controls are defined from several samples and distributed across measurement runs to detect drift in spectral peaks across runs (Wehrens et al., 2016). Replication can be defined at multiple workflow steps, including temporal sampling frequency, biological replication at sampling, or technical replication during extraction or library preparation. Replicates can be processed together in the same batch or deliberately distributed across extraction batches and sequencing runs. Within the same batch, technical replicates quantify baseline measurement variability under nominally identical conditions. In contrast, replication across batches reveals systematic differences in processing and sequencing, which inform downstream batch-effect correction. Longitudinal multiomics studies must allocate samples across timepoints, omics layers, biological replicates, and technical extractions simultaneously, a challenge absent from cross sectional designs and one with no generic solution, since the optimal allocation depends on the planned analysis. For a given budget constraint, power analysis can simulate hypothetical experiments under alternative replication schemes to find the most statistically efficient design (Williams et al., 2020; Sankaran et al., 2024).

### 4.2 Data management

Data management in longitudinal multiomics studies requires careful coordination. Inconsistencies in organization across assays or timepoints can increase the risk of analysis errors. Fortunately, advances in data management software can automatically validate data organization and support quality checks through interactive scripting sessions. In the R Bioconductor software ecosystem, the MultiAssayExperiment (Ramos et al., 2017), TimeSeriesExperiment (Nguyen, 2020), and Legato (Odom, 2024) data structures are notable examples. MultiAssayExperiment coordinates sample indexing and filtering across multiomics layers. When instantiating this data structure, potential naming inconsistencies across component layers are immediately highlighted. This structure streamlines data access so that all layers within a time window can be extracted with a single line of code rather than one line per layer. Similarly, TimeSeriesExperiment and Legato offer additional validation and access management tools for longitudinal data. For example, data stored in a Legato object can be immediately visualized as longitudinal stacked barplots, helping verify the data’s temporal sampling design before analysis.

Reproducibility is particularly challenging in multiomics studies because each biological layer generates its own raw data files and requires distinct preprocessing steps, some of which must be executed remotely on high-performance computing clusters. Data flow managers like Snakemake (Köster and Rahmann, 2012) and targets (Landau, 2021) address these difficulties by systematizing the sequence of data transformations and streamlining their parallel execution. For each workflow step, researchers specify the required input files, expected outputs, and the terminal command and parameters linking them. Since the entire data processing pipeline is specified programmatically, it supports easy reproducibility and protects against human error.

### 4.3 Technical variation

Technical variation refers to data patterns arising from systematic differences in the measurement mechanism rather than underlying biology. For instance, differences in read depth and sampling times can make biologically identical samples appear different. These differences must be accounted for, either by choosing methods aware of potential technical variation or by applying a correction method in advance. Before within-subject temporal dependence is modeled in downstream analyses, addressing technical variation is generally performed at the sample level and relies fundamentally on robust interpolation, normalization, and batch effect correction.

Interpolation is used when samples are not measured at uniformly spaced times, which is a requirement for autoregressive models (see Section 5). Peleg and Borenstein (2024) systematically evaluated interpolation methods across metagenomic datasets with varying sample sizes and temporal sampling designs and found that nearest neighbor interpolation matched or even outperformed more computationally advanced alternatives. For intuition, nearest neighbor interpolation generalizes linear interpolation. Instead of imputing a missing timepoint with a weighted average of the two nearest timepoints, nearest neighbor interpolation imputes the missing timepoint with a weighted average of the *K* nearest timepoints, where the weights are derived from the distances to the timepoint-to-impute. While this method was found to be reliable across metagenomics datasets, it is worth noting that interpolation accuracy can vary across sites/hosts, since less stable microbiomes are more challenging to interpolate. Therefore, site-wise interpolation accuracy should be reported by deliberately reserving some observed times as an interpolation test set.

In sequence-based studies, sequencing depth is a nuisance parameter that influences the observed data but which is not related to hypotheses of interest. A normalization transformation can help place samples on the same scale (Lin and Das Peddada, 2020; Sun and Xia, 2024). In a microbiome context, this can include rarefaction, which downsamples read depths to the shared depth. This discards a large fraction of reads when sequencing depths are uneven (McMurdie and Holmes, 2014). In some cases, it is possible to recover power by generating a consensus across repeated rarefactions, a question studied systematically in (Schloss, 2024; Hong et al., 2022). Aside from rarefaction, it is possible to rescale each sample to make read depths comparable. The most straightforward approach is to convert the original counts to compositions by dividing by total depth. Additional transformations from classical compositional data analysis (Aitchison, 1994; Greenacre, 2021) like the centered log-ratio (CLR) and isometric log ratio (ILR) or microbiome specific variants, like the phylogenetic ILR (Silverman et al., 2017), can then be applied. Note that the initial compositional transformation may be sensitive to outliers – a large count for a single taxon can change the denominator for the entire sample.

More robust alternatives, such as those in (Ma et al., 2020), are considered in the wrench (Kumar et al., 2018) and RioNorm2 packages. While traditional normalization methods are designed for cross-sectional data, leveraging temporal structure can improve normalization quality. For example, TimeNorm (Luo et al., 2024) builds off the insight that, before any interventions have been applied in a time course experiment, there should be no systematic differences across treatment groups, an idea related to specifying a “pool of controls” in more general experimental designs found in omics analysis (Zhang et al., 2025).

Batch effect correction can be especially problematic in longitudinal data, because in long-running experiments, the lab environment itself may change. Changes in sample preparation, storage protocols, or personnel can all influence data generation (Wang and LêCao, 2019). Fortunately, batch effect correction methods have been developed for 16S amplicon, metagenomic, and metabolomic data. Further, their accuracy can be improved by providing known lab environment factors, controls, or replication. In particular, RUVg (Risso et al., 2014; Hardwick et al., 2018), PLSDA-batch (Wang and Lê Cao, 2023), and ConQuR (Ling et al., 2022) are batch effect correction techniques validated on microbiome data. RUVg and PLSDA-Batch are linear models that use latent factors to model batch effects. RUVg and PLSDA-batch both assume data to be normalized in advance, while ConQuR models raw counts directly through a quantile model. RUVg additionally leverages technical replication and features that are assumed to be equivalent across all true biological states. ConQuR similarly includes batch effects as an unobserved factor, but applies a zero-inflated quantile regression model instead of a linear model. This allows the correction to consider batch-induced shifts in the observed features beyond the mean and the variance, as it can explicitly reflect changes in species presence/absence across batches.

Though we have noted some exceptions, like TimeNorm, most normalization and batch-correction methods are designed for independent samples rather than longitudinal data. Since within-subject samples are correlated, their technical (e.g., batch effect) variation can be confounded with per-subject variation. Some batch-correction methods, like ConQuR, RUVg, PLSDA-batch, accept known factors as covariates, allowing us to model time or subject ID directly. Alternatively, randomizing each subject’s timepoints across batches (Section 4) helps to reduce the strength of confounding from the outset. Further, interpolation, normalization, and batch effect correction steps can influence one another, so their order needs to be chosen deliberately, with assumptions checked at each step and sensitivity analysis run on key findings. Batch effect correction methods often estimate each sample’s intrinsic within-batch variation (Leek et al., 2012; Zhang et al., 2025). If interpolation is applied first, it can smooth out real variation before batch effect correction, reducing variance estimates and underestimating the heterogeneity of the original data. Interpolation and normalization interact in a similar way. Spline-based interpolation assume symmetric random errors (Green and Silverman, 1993), an assumption that holds more reliably after transformations, like CLR, that map the data to an unbounded, continuous range.

## 5 Modeling interactions over time and across layers

Microbiome dynamics are the result of interactions across both time and biological layers. Features can influence each other instantaneously (e.g., microbes affecting metabolite levels) or through delayed effects (changes in microbial composition shifting metabolite profiles, which then affect gene expression and functional activity at later timepoints). Modern statistical and machine learning methods can capture both types of interaction, described below and summarized in **Supplementary Table 2**. The discussion here is conceptual – readers seeking mathematical details may consult the “Mathematical Summaries” supplement.

We review both methods designed for cross-section integration and methods that are explicitly time-aware. We discuss cross-sectional methods [e.g., MOFA2 (Argelaguet et al., 2020), DIABLO (Singh et al., 2019), WGCNA (Langfelder and Horvath, 2008), and NetworkAnalyst (Xia et al., 2015)] because longitudinal integration methods are often built from them. For example, MEFISTO (Velten et al., 2022) is a direct time-aware extension of MOFA2. Moreover, these extensions often follow one of a few strategies, like introducing subject-level random effects or deriving interval-specific summaries, detailed below.

### 5.1 Trends and temporal shifts

Temporal patterns can appear at multiple resolutions, from localized shifts in a few features to global trends. Some methods collapse longitudinal data into static summaries. Others model full temporal trajectories, with approaches ranging in complexity depending on their assumptions about how features evolve and interact over time.

Collapsing methods replace each subject’s time course with a single representative sample (Wang et al., 2022), which selects the timepoint with the largest sample size. This discards potentially relevant data. In contrast, treating timepoints as repeated measures makes use of all available data while accounting for within-subject dependence. For example, Fei et al. (2025) proposed a penalized regression model to select taxa associated with patient outcomes. They allow different correlation structures of the longitudinal samples, including “Independence” (no correlation), “Symmetry” (constant correlation), and “Autoregressive” (exponentially decaying correlation). These decisions account for alternative forms of within-subject dependence, but do not model systematic changes in trajectory means. A closely related approach uses mixed-effects modeling, which has been extended to handle sparsity and compositionality in microbiome data (Chen and Li, 2016; Little et al., 2024) and is now implemented in software frameworks like MaAsLin3 (Nickols et al., 2026). However, mixed effects models assume that features have global effects across all timepoints, which might not be realistic. In contrast, interval-specific methods pinpoint when treatment effects occur. For each microbiome feature, measurements can be represented as smooth curves over time. Permutation tests then flag systematic differences between groups within specific time windows (Paulson et al., 2017; Metwally et al., 2022). These approaches accommodate nonuniformly spaced sampling and make no parametric assumptions, but they need sufficiently dense sampling to estimate trends reliably.

When sample sizes or feature dimensions are large, interval-specific testing becomes computationally expensive and hard to interpret. Unsupervised methods address this by focusing on patterns shared across many samples or taxa. Sherwani et al. (2025) found that foundational techniques like *K*-means and Hierarchical Clustering can be successfully applied to a variety of multiomics settings, provided the distance or similarity metric (e.g., Manhattan distance, Bray-Curtis similarity) is chosen to match the data’s structure. Further, trajectories can be clustered, then interpreted with feature enrichment analysis or network visualization (Bodein et al., 2019). Alternatively, continuous latent factors can reflect trajectory variation without hard cluster assignments. In the timeOmics package, Bodein et al. (2022b) identify sparse latent components that define temporal patterns. Each factor involves only a small number of molecular features, simplifying interpretation. Applied to a dataset of Type 2 diabetes progression, the technique identifies predictive trajectory patterns and clarifies which microbes, metabolites, and host RNA molecules change during the disease development process. These approaches have been evaluated for data with few, unevenly spaced timepoints and can learn smooth trends. However, they do not natively return estimates of modeling uncertainty or offer inferential guarantees, so are better suited to exploratory analysis.

Beyond description, generative models can simulate how interventions propagate through a microbiome over time. For example, Ruiz-Perez et al. (2021) designed a dynamic Bayesian network to learn interactions between microbiome and host omics across adjacent time measurements. As a preliminary step, this method interpolates trajectories onto an even grid and aligns them to a reference, a step which rules out alternative normalization methods and is only appropriate when sampling is sufficiently temporally dense. The network structure encodes biological knowledge – specifically, edges flow from current taxa populations to current microbial gene abundances, then to current metabolite abundances, and finally to future taxa populations. These intra- and inter-timepoint edges capture both functional relationships and the temporal dynamics of the system. The learned model can simulate growth curves under varying host and metabolite conditions. Ranking metabolite-taxon interactions by their simulated intervention effects yielded positive and negative interactions that were validated experimentally.

Additionally, Bayesian approaches support modeling of nonlinear trajectories, estimating both population-level patterns and subject-level variation. One example of this is functional principal component analysis (FPCA), which identifies typical modes of variation in the curves. When FPCA is formulated in a Bayesian framework, it improves robustness under sparse and irregularly sampled trajectories and allows flexible prior specification (Jiang et al., 2019, 2022). In particular, this approach models trajectories while bypassing an initial interpolation step. The results are used to estimate the overall mean curves and a set of PC curves for each omics, as a resolution for the temporal associations.

Choosing between methods depends on the target of inference – i.e., the effect the study aims to characterize and expects to generalize to future studies – and on temporal sampling density. If the goal is to identify predictive biomarkers, then collapsing methods or repeated measures methods, are a reliable approach. They ignore complex trajectory shapes but do not require long temporal trajectories. If instead the target is to identify time windows where trends differ, then interval-specific methods are appropriate. These methods also require fewer modeling assumptions, using nonparametric permutation tests rather than analytical formulas. Finally, full generative models can estimate more subtle feature-to-feature relationships and latent trends. However, they require denser sampling and are more sensitive to violations of the assumed data-generating process.

### 5.2 Cross-layer relationship

A central goal of longitudinal multiomics is to clarify how separate biological layers relate to one another over time. This requires extensions of data integration methods to the longitudinal setting. We next summarize the main categories of integration methods and explain the longitudinal variants that have been proposed.

Statistical methods for single-variable predictions are widely available. Therefore, one common approach is to treat one assay as a “response” and fit one regression model per feature within the prioritized assay. Specifically, for each gene in a metatranscriptomic assay, Mallick et al. (2021) learn one linear model whose outcome is measured RNA level and whose predictors are the same gene’s DNA measurement. When there are many candidate features, the number of cross-layer pairs to consider becomes unwieldy. Therefore, it can help to first screen features within each biological layer and only establish cross-layer associations for those screened features. In a longitudinal setting, this filtering can be guided by temporal trends. In such cases, Tan et al. (2024) first identify driver microbes using an autoregressive model. While separate linear regression models are still learned for each metabolite in their data, the predictor set only includes driver microbes. While an autoregressive model supports targeted driver microbe selection, it requires denser temporal sampling and makes stronger assumptions on the relationship between timepoints, ruling out sharp transitions, for example.

Rather than integrating features directly across assays, one can first learn summary scores within each assay then relate these scores across assays or with a long-term outcome. This is a form of data integration through late fusion. For example, Wang et al. (2022) define microbiome risk scores using a feature screening and diversity calculation. This risk score is then combined with polygenic risk scores from other omics in a model of overall risk. This approach exchanges fine-grained temporal modeling for modularity between analysis steps. In an application to predicting Type 1 Diabetes (T1D) risk from the longitudinal TEDDY study, the proposed microbiome risk score is combined with an established polygenic risk score for T1D, yielding a multiomics risk score. Summary scores can also be learned simultaneously across biological layers through intermediate phase data integration. For example MOFA2 defines a joint latent factor model whose generated data can be continuous, binary, or count valued (Argelaguet et al., 2020), and its MEFISTO extension (Velten et al., 2022) adds a Gaussian process component to account for smooth variation over time, like in the infant microbiome development case study featured in the paper and the accompanying software package. Similarly, DIABLO (Singh et al., 2019) and netOmics (Bodein et al., 2022a) integrate multiple biological layers using regularized block partial least squares, one block per layer. Each block is associated with latent factors that capture most of the variation present in that layer. The resulting factors support network visualization: nodes represent features from different omics layers and edges represent strong co-abundance patterns. Note however that both latent factor and partial least squares-based methods require samples to be aligned across modalities and cannot directly be applied to mosaic data, and the DIABLO publication does not provide a microbiome case study, though this has previously been explored (Mujchariyakul et al., 2025). To navigate these multiomics networks, netOmics (Bodein et al., 2022b) used random walks from seed nodes to identify tightly connected feature sets. The temporal structure in netOmics is derived from timeOmics, so these methods complement one another, and a case study from (Bodein et al., 2022b) highlights the recovery of sets of interacting RNA, protein, and cytokine features in longitudinal microbiome data. Alternatively, feature sets can be discovered interactively from larger networks through appropriately defined dynamic queries, like those made available through the NetworkAnalyst interface (Xia et al., 2014, 2015, Zhou et al., 2019a) and its microbiome-specific analog MicrobiomeAnalyst (Chong et al., 2020). To decide between late and intermediate phase integration, consider the benefits of modularity relative to those of a combined model. When temporal sampling is not aligned across layers, then a modular, late-integration approach is preferred, even if it doesn’t explicitly model the relationships between trajectories. However, if sampling is consistent across layers and an initial exploratory analysis suggests shared variation, then an intermediate integration analysis can offer a unified treatment of the data.

Beyond associations, causal methods attempt to clarify the influence of interventions across biological layers. Indeed, the multiomics dynamic Bayesian network (Ruiz-Perez et al., 2021) described above provides a causal interpretation of how changes in one layer propagates across layers and to the next time step. However, this interpretation holds only when the assumed graphical model is correct. Relationships between observed quantities can be checked with posterior predictive checks, but the absence of unmeasured confounders cannot be verified from the data alone. Instead of one-step graphical model links, Zhang et al. (2022) consider cross-layer relationships whose strength is time-scale dependent. The network inference method WGCNA (Langfelder and Horvath, 2008) is applied jointly to features from all omics sources, but before constructing the network, trajectories are smoothed to varying degrees. Associations learned in the more strongly smoothed data are understood as those that are present at long time scales. Causal relationships across layers can also be hypothesized through more specialized mechanisms. For instance, mediation analysis partitions an intervention’s effect into indirect effects mediated by features from other omics layers and a direct effect on a layer of primary interest (Jiang et al., 2025; Qiao et al., 2024). Estimated effects can be biased by unmeasured mediators, so an analysis with these methods should include causal sensitivity analyses, summarizing the strength of unmeasured confounding needed to overturn the study’s conclusions. In each of these cases, the temporal sampling design support causal analysis that generate mechanistic hypotheses about how molecular layers interact with one another. However, these models also assume the absence of unmeasured confounders and can fail under model misspecification. Model checks are an especially important part of their analysis workflow. Even a causal model should be viewed as hypothesis generating, rather than definitive proof of a biological mechanism.

## 6 Discussion

Statistical methods for longitudinal multiomics analysis can uncover trends and associations that are invisible in other sampling designs. While care must be taken to account for potential technical artifacts, the results from these multiview studies can guide specific experimental follow-up. For example, metabolite-microbiome interactions can be recapitulated in organ-on-a-chip experiments (Mahajan et al., 2022), and microbe-microbe interactions can be analyzed using model communities (Lozano et al., 2019).

Microbiome biomarker construction can often be framed as a phenotype prediction problem, making it amenable to accuracy benchmarking on holdout test sets. In contrast, temporal and cross-layer relationships cannot be validated using out-of-sample prediction alone. Instead, assessments can be based on the stability of the learned associations to perturbations of the input data, preprocessing judgment calls, and modeling hyperparameters (Yu and Kumbier, 2020). Simulation experiments can lend further credibility to analysis workflows (Tonkin-Hill, 2023; Ma et al., 2021; Song et al., 2023). Comparison across studies and reproducibility can be enhanced using public data repositories and preparation of appropriate software modules.

New data modalities like spatial (Lötstedt et al., 2023; Ntekas et al., 2024) and single-cell (Zheng et al., 2022; Lloréns-Rico et al., 2022) microbial profiling will continue to emerge, and existing assays will become cheaper and more accessible. The methodological community will also continue exploring novel classes of models, including those based on Bayesian trajectory analysis (Jiang et al., 2022; Gygi et al., 2024), state space models (Xu et al., 2023), neural ordinary differential equations (Borsari et al., 2025; Thompson et al., 2026), deep generative models (Brombacher et al., 2022), and transformer-based architectures (Liu and Park, 2024). Nonetheless, the principles behind effective longitudinal multiomics analysis – like careful experimental design, structured data management, and interaction modeling – will carry over, even as novel methods adapt to new assay types. Together, these ideas can support a view of microbial communities that faithfully captures the richness of their interactions and potential for change.
