# Supplementary material for: Design, processing, and modeling for longitudinal multiomics microbiome data
Source: Front Cell Infect Microbiol. 2026 Aug 20;16:1837109. doi: 10.3389/fcimb.2026.1837109 (PMC13539529; doi:10.3389/fcimb.2026.1837109)
Supplement: Supplementary file 1 [file Supplementaryfile1.pdf]

SUPPLEMENTARY TABLE

Table 2. Summary of research objectives, approaches, and methods discussed in Section 5. **Approaches** define how methods account for read depth: *Log-ratio based* models leverage compositional ratios; *Count model based* approaches handle raw counts directly; *User-specified* methods allow for flexible pre-transformation. **Characteristics** are shown in Fig 1 and defined by inferential target (**C1**: Differential Trajectory Analysis; **C2**: Cross-Layer Covariation; **C3**: Inter-layer Causal Propagation), data representation (**D1**: Collapsed representation; **D2**: Repeated measures; **D3**: Trajectories and trends; **D4**: Generative transitions), and temporal sampling requirement (**S1**: requires regular sampling or interpolation on a common grid; **S2**: supports irregular sampling directly).

Research Objective	Approach	Method	Bibliography	Char.	Phase
Temporal Trends and Shifts	Log-ratio based	MRS framework	[Wang et al., 2022]	D1, –, –	–
	Log-ratio based	FLORAL	[Fei et al., 2025]	C1, D2, S2	–
	Log-ratio based	MaAsLin3	[Nickols et al., 2026]	C2, D2, S2	Late
	User-specified	cov-adj PCoA	[Little et al., 2024]	C1, D2, S2	–
	User-specified	metaSplines	[Paulson et al., 2017]	C1, D3, S2	–
	User-specified	OmicsLonDA	[Metwally et al., 2022]	C1, D3, S2	–
	Count model	DBN	[Ruiz-Perez et al., 2021]	C3, D4, S1	Early
	User-specified	Bayesian FPCA	[Jiang et al., 2022]	C1, D3, S2	Intermediate
Cross-Layer Relationships	Log-ratio based	MaAsLin 2	[Mallick et al., 2021]	C2, D2, S2	Late
	User-specified	mbDriver	[Tan et al., 2024]	C1, D4, S1	Late
	User-specified	late fusion	[Wang et al., 2022]	C2, D1, –	Late
	User-specified	DIABLO	[Singh et al., 2019]	C2, D1, –	Intermediate
	User-specified	NetworkAnalyst	[Xia et al., 2014]	C2, –, –	Late
	User-specified	WGCNA	[Zhang et al., 2022]	C3, D3, –	Early
	Count model	mediation	[Jiang et al., 2025]	C3, D2, S2	Late
	Count model	MOFA2	[Argelaguet et al., 2020]	C2, D1, –	Intermediate
Both (Trends and Cross-Layer)	User-specified	timeOmics	[Bodein et al., 2022b]	C1, D3, S2	Intermediate
	Count model	MEFISTO	[Velten et al., 2022]	C2, D3, S2	Intermediate
	User-specified	netOmics	[Bodein et al., 2022a]	C2, D3, S2	Late

MATHEMATICAL SUMMARIES

Trends and temporal shifts

Shared Notation

Indices

- $i = 1, \ldots, n$: subject index.

- $j = 1, \ldots, m_i$ or $t = 1, \ldots, T_i$: within-subject sample or time index.

Data

- Y_{ij} or $Y_i(t)$: outcome / response for subject i, sample j or time t.
- $X_{ij} \in \mathbb{R}^p$: microbial feature vector (counts or relative abundances).
- $W_{ij} \in \mathbb{R}^L$: subject covariates.

Model Components

- θ: parameter vector.
- $g(\cdot)$: link function.
- λ: regularization parameter.

SS-ANOVA splines for longitudinal differential abundance [Paulson et al., 2017]

The kth replicate of subject i's trajectory at sample j (measured at time t) is assumed to follow,

$$X_{ijk} = \gamma^\top W_i + f_t(t) + f_g\left(\mathbf{1}\{i \in g\}\right) + f_{tg}\left(t, \mathbf{1}\{i \in g\}\right) + \varepsilon_{ijk}, \qquad \varepsilon_{ijk} \sim \mathcal{N}(0, \sigma^2).$$

where f_t is the smooth main effect over time, f_g the group g main effect, f_{tg} the time-by-group interaction, and $\mathcal{H}$ is an RKHS. These parameters are estimated using a penalized regression,

$$\min_{f \in \mathcal{H}} \sum_{i,j,k} \big(X_{ijk} - f(\cdot)\big)^2 + \lambda J(f)$$

where J penalizes the complexity of the spline trajectory f. The penalty λ is chosen using a variant of generalized cross-validation. A test statistic is defined as the area A_{t_1,t_2} between group-specific trajectories over intervals of large group difference,

$$A_{t_1,t_2} = \int_{t_1}^{t_2} \widehat{D}_g(t)dt$$

where group differences $D_{g(t)}$ and intervals I_{t_1,t_2} are defined as,

$$\begin{aligned} D_g(t) &= f_g(1) + f_{tg}(t, 1) \\ I_{t_1,t_2} &= \big\{t \in [t_1, t_2] : \widehat{D}_g^L(t) \leq -\eta \text{ or } \widehat{D}_g^U(t) \geq \eta\big\}. \end{aligned}$$

Significance is evaluated by permuting the group label $\mathbf{1}\{i \in g\}$ across subjects B times, recomputing $A^b_{t_1,t_2}$, and define an empirical p-value as the number of times the permuted $A^b_{t_1,t_2}$ exceed the statistic on observed data.

FLORAL: penalized log-ratio GEE for longitudinal outcomes [Fei et al., 2025]

The long-term clinical outcome Y_{ij} is related to microbial features X_{ij} and other clinical covariates W_{ij} through parameters $\theta = \left(\beta^\top, \gamma^\top\right)^\top$ and link function g,

$$\mathbb{E}[Y_{ij}] = g\left(\sum_{k=1}^{p} \beta_k \log X_{ij,k} + \sum_{l=1}^{L} \gamma_l W_{ij,l}\right)$$
$$\text{Var}(Y_{ij}) = \phi v_{ij}(\theta)$$

subject to the constraint $\sum_{k=1}^{p} \beta_k = 0$ and variance terms v_{ij} are derived from the working correlation on Y_{ij}. Parameters β_k and γ_l can be estimated using the classic generalized estimating equation framework with estimating function

$$S(\theta; \phi, \rho) = \sum_{i=1}^{n} \left(\frac{\partial \mu_i}{\partial \theta}\right)^\top V_i^{-1}\big(Y_i - \mu_i(\theta)\big) = 0$$
$$\text{s.t. } \sum_{k} \beta_k = 0.$$

To handle high-dimensionality, a regularized form of this equation is used during the optimization for θ,

$$U(\theta) = S(\theta; \widehat{\phi}, \widehat{\rho}) - n \begin{pmatrix} q_{\lambda_\beta}(|\beta|) \odot \text{sign}(\beta) - \mathbf{1}_p \left(\eta \sum_k \beta_k\right) \\ q_{\lambda_\gamma}(|\gamma|) \odot \text{sign}(\gamma) \end{pmatrix}$$

where q_λ are penalty functions, like the ℓ^1 penalty, that encourage lower-complexity fits.

ZIBR Model: Zero-Inflated Beta Regression with Random Effects [Chen and Li, 2016]

This model considers each taxon l separately. Hence, to simplify presentation, we overload notation to focus on the relative abundance of taxon l: $X_{it} := X_{itl}$. Covariates are split into blocks $W_{it}^{(1)}$ and $W_{it}^{(2)}$ driving presence absence and nonzero abundance, respectively. The observed relative abundance is modeled as,

$$X_{it} = \begin{cases} 0 & \text{w.p. } 1 - p_{it} \\ \text{Beta}(\mu_{it}\phi, (1 - \mu_{it})\phi) & \text{w.p. } p_{it} \end{cases}$$

where the presence probability and beta mean are influenced by both covariates and subject-specific random effects,

$$\text{logit}(p_{it}) = \gamma_0^{(1)} + (W_{it}^{(1)})^\top \gamma^{(1)} + a_i, \qquad a_i \sim \mathcal{N}(0, \sigma_a^2)$$
$$\text{logit}(\mu_{it}) = \gamma_0^{(2)} + (W_{it}^{(2)})^\top \gamma^{(2)} + b_i, \qquad b_i \sim \mathcal{N}(0, \sigma_b^2).$$

The likelihood of $\gamma^{(1)}, \gamma^{(2)}$ marginalizing out the random effects can be maximized using Gauss-Hermite quadrature. Likelihood ratio tests can be derived for the null hypotheses

$$H_0^{\text{presence}} : \gamma_j^{(1)} = 0$$
$$H_0^{\text{abundance}} : \gamma_j^{(2)} = 0$$
$$H_0^{\text{both}} : \gamma_j^{(1)} = \gamma_j^{(2)} = 0$$

Bayesian sparse functional PCA [Jiang et al., 2022]

Represent the trajectory for a single taxon as,

$$Y_i(t) = \mu(t) + \sum_{r=1}^{K} f_r(t)\alpha_{ir} + \varepsilon_i(t)$$

with orthonormal eigenfunctions $\int f_r f_s = \delta_{rs}$ and scores $\alpha_i \sim \mathcal{N}(0, \text{diag}\left(\sigma_\alpha^2\right))$. Each index r is interpreted as a separate functional principal component dimension. Stacking across taxa and expanding μ_i and f_r in a cubic spline basis, the discrete-time multivariate version is

$$Y_i = B_i\theta_\mu + B_i\Lambda\alpha_i + \varepsilon_i \qquad \varepsilon_i \sim \mathcal{N}(0, \sigma_\varepsilon^2 I)$$

where B_i and Λ are block diagonal matrices derived from the spline basis. Placing appropriate priors over θ_μ, $\Lambda_{\cdot,r}$, and σ_ϵ the model can be estimated using Hamiltonian Monte Carlo using the Stan probabilistic programming language. Note that Λ is only identifiable up to orthogonal rotations. During MCMC, Λ is sampled freely but rotated post hoc according to the top K eigenvectors during each MCMC iteration. Further, the number of functional principal components is chosen according to the PSIS-LOO out-of-sample likelihood evaluation technique.

Spline-based partial least squares integration [Bodein et al., 2019]

This approach has three main steps: per-feature spline modeling, clustering of fitted trajectories, and multiblock integration.

In the first step, consider a series of increasingly complex models and choose the simplest with acceptable fit to the data,

1. Linear regression: $Y_{it} = \theta_0 + \theta_1 t + \varepsilon_{it}$.
2. Random-intercept: $Y_{it} = (\theta_0 + b_{0i}) + \theta_1 t + \varepsilon_{it}$.
3. Random intercept + slope: $Y_{it} = (\theta_0 + b_{0i}) + (\theta_1 + b_{1i})t + \varepsilon_{it}$.
4. Random effects cubic spline: $Y_{it} = \theta_0 + \sum_r \theta_r B_r(t) + b_{0i} + \varepsilon_{it}$ estimated with smoothness-encouraging penalty $\sum_r \theta_r^2$.

For each omics block q, combine the model fitted values into matrix $Z^{(q)} \in \mathbb{R}^{T \times P_q}$. This gives prototypical trajectories for each feature across all subjects.

In the clustering step, decompose each $Z^{(q)}$ using PCA. The associated scores are clustered with the choice of K governed by the silhouette index. In the final integration step, solve a multiblock canonical

correlation problem,

$$\max_{a_h^{(1)},\ldots,a_h^{(Q)}} \sum_{q\neq r} c_{qr}\mathrm{cov}\big(Z_h^{(q)}a_h^{(q)}, Z_h^{(r)}a_h^{(r)}\big) \quad \text{s.t. } \|a_h^{(q)}\|_2 = 1, \|a_h^{(q)}\|_1 \leq \lambda^{(q)} \tag{1}$$

where the c_{qr} are user-specified parameters controlling how blocks are related.

Multiomics dynamic bayesian network [Ruiz-Perez et al., 2021]

The first defines a DAG $\mathcal{G}$ reflecting hypothesized causal relationships between microbial genes, metabolites, host genes, and environmental covariates. Denote the parents of node $v \in V$ by $\mathrm{Pa}_{\mathcal{G}}(v)$. Then, the likelihood of consecutive time steps is given by

$$p(V) = \prod_{v\in V} f\left(v|\mathrm{Pa}_{\mathcal{G}}(v)\right).$$

When features are continuous, a linear Gaussian is used,

$$f(v \mid u_1,\ldots,u_K) = \mathcal{N}\left(\theta_0 + \sum_{k=1}^{K}\theta_k u_k, \sigma^2\right).$$

To ensure interpretable results, the input trajectories are first smoothed for each subject, interpolated onto an even grid, and aligned to a reference trajectory via linear time warping. The graph $\mathcal{G}$ and model parameters Θ are estimated using an alternating optimization over the likelihood,

$$\max_{\Theta,\mathcal{G}} P(D \mid \Theta,\mathcal{G})P(\Theta,\mathcal{G}) \tag{2}$$

where in the $\mathcal{G}$-optimization step the choice of edges is greedily chosen to maximize the improvement in the Bayesian Information Criterion.

MRS: Microbial Risk Score [Wang et al., 2022]

The samples $X_1,\ldots,X_n$ represent the microbiome relative abundances across $(\text{subject}, \text{time})$ pairs. In particular, longitudinal structure is not explicitly modeled. The procedure operates in two steps analogous to polygenic risk scoring in genome wide associations studies. In the first signature identification step,

1. Run ANCOM-BC for differential abundance testing in a discovery cohort.
2. Pick taxa sets $S(\pi)$ at increasing p-value thresholds π.
3. For each $S(\pi)$, define a candidate microbial risk score (MRS).
4. Choose π^* maximizing AUC and set $S^* = S(\pi^*)$.

Step 2 refines the candidate subcommunity into a final MRS by renormalizing within the subcommunity. Specifically, for $k \in S^*$, let

$$\widetilde{X}_{ik} = \frac{X_{ik}}{\sum_{k'\in S^*} X_{ik'}} \tag{3}$$

and compute the negative alpha-diversity, so that smaller MRS corresponds to healthier communities,

$$\mathrm{MRS}_{\alpha}^{(i)} = \sum_{k=1}^{|S^*|} \widetilde{X}_{ik} \ln \widetilde{X}_{ik}. \tag{4}$$

While this is a sample-level score, it can be incorporated into the analysis of longitudinal cohorts through a temporal outcome model (e.g., survival analysis).

Cross-layer relationships

MaAsLin 2 [Mallick et al., 2021]

As in [Chen and Li, 2016], MaAsLin 2 models each taxon l separately, so we similarly denote $X_{ij} := X_{ijl}$ for the remaining discussion. Further, we suppose that the counts X have been normalized into compositions in advance. The recommended default is a linear mixed model on log-transformed compositions,

$$\begin{aligned}\log\left(X_{ij} + c\right) &= \gamma_0 + W_{ij}^{\top}\gamma + u_i + \varepsilon_{ij} \\ u_i &\sim \mathcal{N}(0, \sigma_u^2) \\ \varepsilon_{ij} &\sim \mathcal{N}(0, \sigma^2),\end{aligned}$$

where c is a pseudocount to avoid $\log(0)$ and u_i is a random effect to capture within-subject dependence. Further, the package supports several link functions, which correspond to different data transformations,

1. *Linear*: Log-transformed, pseudocount-adjusted compositions.
2. *Negative binomial*: Raw counts with overdispersion.
3. *Compound Poisson*: Zero-inflated, continuous positive data, like relative abundances without a pseudocount.
4. *Zero-inflated Negative Binomial*: Counts with excess zeros.
5. *Logistic*: Presence/absence data.

In each case, the predictor $W_{ij}^{\top}\gamma + u_i$ is passed through an appropriate link function, and estimation is accomplished by maximum or restricted maximum likelihood for each feature.

MOFA+ [Argelaguet et al., 2020]

MOFA is a latent factor model generalized to M omics layers and G sample groups (e.g., batches or treatment conditions). For modality m and group g with N_g samples and D_m features, it assumes the linear factorization for the observations $X^{gm} \in \mathbb{R}^{N_g \times D_m}$,

$$X^{gm} = Z^{g}(W^{m})^{\top} + \varepsilon^{gm}.$$

The same factors appear across all modalities for group g, and the same loadings appear across all groups for modality m. This shared structure unifies the factors across layers and groups.

The model is regularized through priors on W^m and Z^g. An automatic relevance determination prior with variance $\frac{1}{\alpha_k^m}$ on the kth column of W^m and with variance $\frac{1}{\alpha_k^g}$ on the kth column of Z^g allows entire factors to shrink to zero with a modality or group. This means that factors can be selectively active, depending on the omics layer and group context. Further, a spike-and-slab prior on entries of W^m and Z^g encourages

sparsity, so that each factor only depends on a sparse subset of omics features and each sample only depends on a sparse subset of factors.

The overall model is fit using stochastic variational inference, which allows estimation over minibatches of data and supports scalability. After fitting, the variance explained by factor k in group g and modality m is given by one minus the unexplained variance,

$$R^2_{gmk} = 1 - \frac{\sum_{n,d}\left(X^{gm}_{n,d} - Z^g_{:,k}(W^m_{:,k})^\top\right)^2}{\sum_{n,d}(X^{gm}_{n,d})^2}$$

DIABLO [Singh et al., 2019]

For each latent component $k = 1, \dots, K$, this model learns loadings $a_k^{(m)}$ that maximize the constrained objective,

$$\max_{a_k^{(1)},\dots,a_k^{(M)},a_k^{(Y)}} \sum_{q \neq r} c_{qr}\,\mathrm{cov}\left(X_k^{(q)}a_k^{(q)},\ X_k^{(r)}a_k^{(r)}\right) \quad \text{s.t. } \|a_k^{(q)}\|_2 = 1,\ \ \|a_k^{(q)}\|_1 \leq \lambda^{(q)}\ \ \forall q,$$

where the indices q, r range over the $M+1$ blocks $\{X^{(1)}, \dots, X^{(M)}, Y\}$. The matrix $X_k^{(m)}$ is a version of $X^{(m)}$ after residualizing out components $1, \dots, k-1$,

$$X_k^{(m)} = X_{k-1}^{(m)} - X_{k-1}^{(m)}a_{k-1}^{(m)}\left(a_{k-1}^{(m)}\right)^\top$$

with $X_0^{(m)} := X^{(m)}$. The ℓ^1 and ℓ^2 constraints encourage sparsity and stability in the loadings. When $C = 0$, the blocks are only linked to Y, not to each other; this encourages predictive accuracy but does not encourage factors to be similar across blocks. Conversely, when $C = 1 - I$, every pair of blocks is connected, and large inter-block correlation is prioritized, even if it reduces predictive accuracy.

WGCNA [Langfelder and Horvath, 2008]

WGCNA is a foundational omics network construction method. Although it is developed for a single omics layer, it has been extended to multiple layers as discussed in the main text. In the first step, the method constructs the absolute or signed correlations between omics features using all N observations. Specifically, rescale features $x_k, x_l \in \mathbb{R}^N$ to the range $[0, 1]$,

$$s_{kl} = |\mathrm{cor}(x_k, x_l)| \quad \text{or} \quad \left|\tfrac{1+\mathrm{cor}(x_k,x_l)}{2}\right|.$$

and reweight to emphasize larger correlations,

$$a_{kl} = s_{kl}^{\beta}, \qquad \beta \geq 1$$

To identify tightly interconnected modules, we convert a_{kl} to a dissimilarity measure that can be passed to hierarchical clustering. Cutting the hierarchical clustering tree yields Q clusters, and the top principal component E^q of the submatrix of X formed by the cluster q features serves as a representative profile for those features. The significance of a module in explaining variation in a trait T is determined by cor (E^q, T).
